# Routinely Available Inflammatory Biomarkers Are Not Associated With Target Lesion Revascularization After Coronary Intervention

**DOI:** 10.1016/j.jacadv.2024.101452

**Published:** 2025-01-22

**Authors:** Eva Steinacher, Andreas Hammer, Ulrike Baumer, Felix Hofer, Niema Kazem, Max Lenz, Irene Lang, Christian Hengstenberg, Patrick Sulzgruber, Lorenz Koller, Alexander Niessner

**Affiliations:** Department of Internal Medicine II, Division of Cardiology, Medical University of Vienna, Vienna, Austria

**Keywords:** acute stent thrombosis, C-reactive protein, inflammation, leukocytes, percutaneous coronary intervention, target lesion revascularization

## Abstract

**Background:**

Biomarkers of inflammation are reliable predictors of adverse cardiovascular events in coronary artery disease. However, the association between systemic inflammation and percutaneous coronary intervention (PCI)-related adverse events remains widely unclear.

**Objectives:**

The objective of this study was to investigate the association of routinely assessed inflammatory biomarkers C-reactive protein, leukocytes, and neutrophil-to-lymphocyte ratio (NLR) with patient outcomes in an unselected patient cohort undergoing PCI.

**Methods:**

A total of 7,412 patients (median age 64 years, 73.2% male) were enrolled in this single-center observational trial with a median follow-up time of 4.6 years. Target lesion revascularization (TLR) and acute stent thrombosis (ST) were defined as primary endpoints. Further endpoints included mortality, cardiovascular mortality, and 3-point major adverse cardiovascular events (MACE) (composite of cardiovascular mortality, myocardial infarction, and stroke). Cox proportional hazard regression was used for statistical analysis with a level of significance set at *P* < 0.01.

**Results:**

In the total study cohort, patients experiencing subsequent TLR (n = 488, 6.6%) had more comorbidities and underwent more complex primary interventions. Interestingly, no relation was found between systemic inflammation and subsequent TLR (eg, adjusted HR for C-reactive protein: 0.93 [95% CI: 0.85-1.03], *P* = 0.150), 30-day TLR (0.89 [95% CI: 0.75-1.05], *P* = 0.177), or acute ST (1.06 [95% CI: 0.79-1.42], *P* = 0.708) in multivariable analysis, neither in elective nor in acute interventions. All investigated inflammatory biomarkers were, however, significantly associated with all-cause mortality, cardiovascular mortality, and 3-point MACE, even after comprehensive adjustment for clinical and interventional parameters.

**Conclusions:**

While our results emphasize the importance of systemic inflammatory activation for mortality and MACE, elevated baseline inflammatory parameters show no correlation with TLR or acute ST and, therefore, should not delay PCI in the era of second-generation drug-eluting stents.

Coronary artery disease (CAD) is among the leading causes of morbidity and mortality on a global scale. Due to the aging society, improved survival rates, and corresponding increased treatment periods, the demands on patient care are high. Along with drug therapy, percutaneous coronary intervention (PCI) is the primary treatment for symptomatic patients. Possibilities of PCI have greatly expanded by constant technical improvements during the last decades of interventional cardiology.^1^ Nevertheless, target lesion revascularization (TLR) due to in-stent restenosis and stent thrombosis (ST) remain a present, potentially life-threatening complication. Inflammatory activation induced by vessel wall response may play a central role in their pathogenesis.^2^

Inflammation, characterized by immune cell activation and release of immune mediators, is a major pathophysiologic pathway in the development and progression of atherosclerosis. Chronic inflammation leads to endothelial dysfunction, lipid accumulation, and consequently plaque formation in the arterial wall. In addition, inflammation is a crucial trigger for thrombosis by inducing procoagulant factors, altering the vessel wall, and promoting platelet aggregation.^3^

Systemic inflammation is routinely evaluated by laboratory parameters with C-reactive protein (CRP) as a key biomarker and principal downstream mediator of the acute-phase reaction. CRP, synthesized in the liver by stimulation of inflammatory cytokines, escalates up to 1,000-fold in acute inflammation. While absent in healthy vessel walls, it accumulates in diseased tissue, activating both the classical and alternative complement pathways, contributing to endothelial dysfunction, plaque instability, and platelet aggregation.^4^ Leukocytes, further routinely assessed inflammatory biomarkers, are activated and recruited by endothelial dysfunction and migrate into the damaged arterial wall. In conjunction with lipoproteins, they trigger further inflammatory responses that contribute to plaque growth.^5^ In particular, the neutrophil-to-lymphocyte ratio (NLR) is considered a reliable and easily accessible marker for subclinical inflammation with prognostic relevance in cardiovascular diseases.^6^^,^^7^

PCI induces a local inflammatory reaction in the vessel wall. While bare metal stents (BMS) and first-generation drug-eluting stents (DES) were still associated with numerous stent-related adverse events, new techniques in drug processing and polymer coating have achieved clinical and angiographic improvements.^8^, ^9^, ^10^, ^11^ While increased inflammatory biomarkers are reliable predictors of adverse cardiovascular events in primary and secondary prevention of CAD, the role of systemic inflammation in PCI and particular intervention-related adverse events is not fully understood. Some studies suggest that clinical and angiographic short-term and long-term outcomes are associated with systemic inflammation in PCI.^12^, ^13^, ^14^, ^15^, ^16^ However, these studies are primarily focused on BMS and early-generation DES and are underpowered due to their small patient cohorts.

Therefore, the primary objective of this study is to investigate whether systemic inflammation, represented by CRP, leukocytes, and NLR, is associated with TLR and early ST. Our secondary aim is to examine the association between the markers of systemic inflammation and mortality and major adverse cardiovascular events (MACE), a composite of cardiovascular mortality, myocardial infarction, and stroke.

## Methods

### Patient population

**Figure 1:**
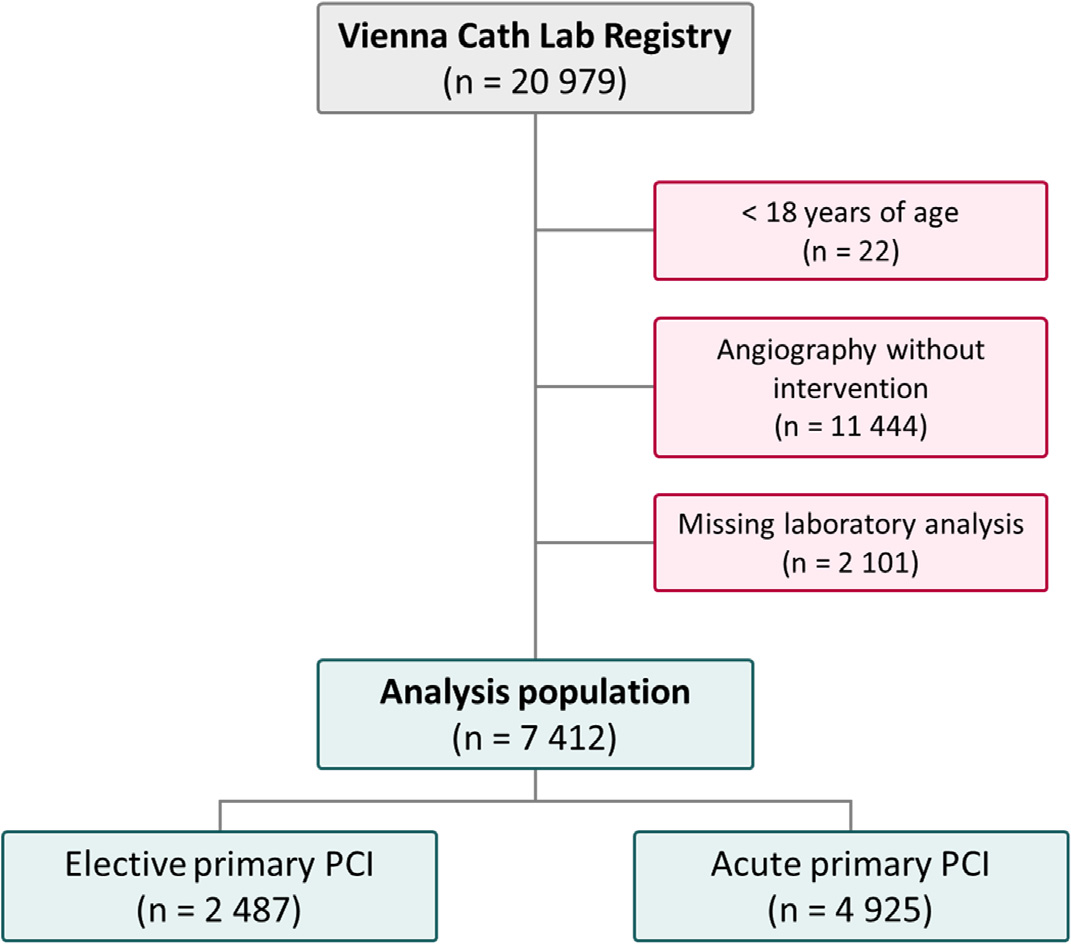
**Flow Chart of the Study Cohort** PCI = percutaneous coronary intervention

The total study cohort comprised 20,979 patients undergoing coronary angiography at a university-affiliated, high-volume cardiac catheterization laboratory at the Vienna General Hospital (Austria) between January 2010 and December 2021. Only patients with a full standard laboratory analysis within 48 hours prior to angiography were included. In selecting the patient population for this study, only individuals undergoing PCI with stent implantation, the use of drug-coated balloons (DCBs), or plain old balloon angioplasty (POBA) were included. Patients under 18 years of age were excluded. The final cohort of this single-center observational study comprised 7,412 patients (Figure 1). NLR data were available in 5,916 individuals due to missing laboratory analysis, as differential blood count including neutrophils and lymphocytes is at the discretion of the admitting physician and therefore not available for each patient. The study protocol is in accordance with the ethical principles of the Declaration of Helsinki and was approved by the ethics committee of the Medical University of Vienna (EK 2043/2021).

### Data acquisition

Our study is based on patient data from the Vienna Cath Lab Registry, which comprehensively captures clinical and angiographic characteristics of patients from the cardiac catheterization laboratory. All variables included in the registry are collected according to precise specifications via the local electronic patient system of the Vienna General Hospital (Austria) and managed in a scientific patient database of the Medical University of Vienna (Austria).

Patients’ baseline characteristics, including age, sex, body mass index (BMI), and previous patient history, were determined close to or at the time of their presentation in the cardiac catheterization laboratory. Routine laboratory analyses were assessed prior to angiography. In addition, data concerning angiographic characteristics such as indication for intervention, coronary anatomy, location and type of treatment, used material, duration of intervention, and contrast volume were obtained from the cardiac catheterization reports.

### Study definitions and endpoints

For categorical analyses, inflammatory laboratory parameters were divided into clinically relevant cut-off values. For CRP, the following categories were chosen: 1) 0 to 0.5 mg/dL (n = 4,337); 2) >0.5 to 1.0 mg/dL (n = 1,213); 3) >1.0 to 2 mg/dL (n = 741); and 4) >2 mg/dL (n = 1,121). Leukocytes were divided into 3 classes: 1) 0 to 10 g/L (n = 4,363); 2) >10 to 12 g/L (n = 1,215); and 3) >12 g/L (1,834). NLR was categorized as follows: 1) 0 to 3 (n = 2,947); 2) >3 to 6 (n = 2,053); and 3) >6 (n = 916). As a measure of global stent size, we calculated a stent ratio defined as the quotient of total stent length and minimal stent diameter.

TLR was defined as the primary measure of an unfavorable interventional outcome. According to the Vienna Cath Lab Registry, TLR was determined as any subsequently performed intervention including stenting, DCB, POBA, thrombus aspiration, or rotablation in the same coronary segment. In addition, reports of all registered TLR that were registered within 30 days after primary intervention were specifically screened for acute ST, and angiographic films were reviewed in case of uncertainty. Further endpoints included mortality, cardiovascular mortality, and 3-point MACE (a composite of cardiovascular mortality, myocardial infarction, and stroke). In order to record endpoints as completely as possible, diagnoses from all hospitals in the Viennese Hospital Association, providing the majority of local patient care, were collected. Dates and causes of mortality were validated by the nationwide Austrian death register (Statistics Austria). Standardized follow-up was carried out until September 2022 via the local electronic patient system.

### Statistical analysis

Data analysis was conducted using SPSS Statistics 27 program (IBM SPSS-Statistics). Due to the large sample size, a 2-sided *P* value of <0.01 was considered statistically significant to reduce the likelihood of type I errors. In case of missing values, cases were excluded from analyses according to listwise deletion.

Categorial parameters are shown as counts and percentages; continuous variables are described as median (IQR). Chi-squared or Fisher’s exact test were used where appropriate for the comparison of event rates between groups. All metric variables were tested graphically by Q-Q plots and histograms for normal distribution. Univariable regression analyses were used to compare baseline characteristics between the TLR and non-TLR cohorts. To investigate the impact of inflammatory biomarkers on the defined primary and secondary endpoints, Cox proportional hazard regression analysis was performed. Proportional hazard assumptions were tested visually by log-minus-log survival curves. Variables not normally distributed underwent logarithmic transformation before entering the model to ensure linearity of continuous variables. In the time-to-event analysis for TLR, deaths were treated as censored events corresponding to a cause-specific hazard model. Results are presented as HR per 1 SD increase in the raw biomarker levels, without prior standardization, along with their 95% CIs. The multivariable regression model was adjusted for age, sex, BMI, indication for intervention, previous revascularization, hypertension, hyperlipidemia, diabetes mellitus type II, glomerular filtration rate (chronic kidney disease-epidemiology collaboration), coronary dominance, SYNTAX score, type of intervention, left main intervention, number of coronary vessels treated, number of coronary vessels diseased, duration of intervention, and contrast volume. Kaplan-Meier charts were plotted to estimate event probabilities according to categories of biomarkers and analyzed with the log-rank test.

## Results

### Baseline characteristics

Demographic and clinical characteristics of the interventional study population (n = 7,412) are presented in Table 1. The median age was 64 years (IQR: 55-74 years), the median BMI was 27.1 kg/m^2^ (IQR: 24.5-30.5 kg/m^2^) and 5,428 (73.2%) patients were male. Stratification into patients with favorable interventional outcome (n = 6,924; 93.4%) and patients with subsequent TLR (n = 488; 6.6%) indicated comparable age, sex, and BMI. However, patients with TLR presented with more comorbidities including previous revascularization, arterial hypertension, hyperlipidemia, or diabetes mellitus type II. In addition, they underwent more complex primary interventions in terms of a higher SYNTAX score, higher number of left main interventions, more severe coronary vessel disease, longer duration of intervention, and the use of more contrast volume. Preintervention laboratory values were similar in both groups, except for infection parameters, which appear to be lower in the TLR group. In case of PCI with stenting, TLR was associated with a higher number of stents and a higher stent ratio.

**Table 1:** Demographic and Clinical Baseline Characteristics

	Total (N = 7,412)	TLR (n = 488, 6.6%)	No TLR (n = 6,924, 93.4%)	*P* Value
Patient characteristics				
Age, y	64 (55-74)	65 (56-73)	64 (54-74)	0.010
Sex				0.118
Male	5,428 (73.2%)	376 (77.0%)	5,052 (73.0%)	
Female	1984 (26.8%)	112 (23.0%)	1,872 (27.0%)	
BMI, kg/m^2^	27.1 (24.5-30.5)	27.3 (24.8-30.5)	27.1 (24.5-30.5)	0.821
Comorbidities				
Previous revascularization	2,323 (31.3%)	220 (45.1%)	2,103 (30.4%)	**<0.001**
Hypertension	3,903 (52.7%)	310 (63.5%)	3,593 (51.9%)	**<0.001**
Hyperlipidemia	2,568 (34.6%)	229 (46.9%)	2,339 (33.8%)	**<0.001**
Diabetes mellitus type II	1,561 (21.1%)	143 (29.3%)	1,418 (20.5%)	**<0.001**
Laboratory analyses				
Hemoglobin, g/dL	13.9 (12.5-15.0)	13.8 (12.4-15.0)	13.9 (12.5-15.0)	0.013
Thrombocytes, g/L	230 (190-276)	218 (188-266)	231 (190-277)	0.029
Leukocytes, g/L	9.18 (7.11-11.96)	8.29 (6.42-10.60)	9.26 (7.16-12.06)	**<0.001**
CRP, mg/dL	0.37 (0.15-1.01)	0.30 (0.12-0.81)	0.38 (0.15-1.02)	0.141
NLR	3.00 (2.08-4.65)	2.68 (1.97-4.19)	3.06 (2.09-4.67)	0.115
NT-proBNP, pg/mL	433.3 (116.9-1690.5)	390.2 (130.9-1359.0)	436.1 (116.1-1713.5)	0.106
Troponin T, ng/L	91 (27-409)	62 (21-369)	92 (28-410)	0.220
LDL, mg/dL	99 (72-130)	95 (67-124)	99 (72-130)	**<0.001**
GFR (CKD-EPI), mL/min	75.9 (57.0-91.3)	73.0 (57.4-89.9)	76.1 (57.0-91.4)	**<0.001**
HbA1c, %	5.8 (5.4-6.3)	5.9 (5.5-6.5)	5.8 (5.4-6.3)	0.066
Percutaneous coronary intervention				
Indication for intervention				**<0.001**
Elective	2,487 (33.6%)	222 (45.5%)	2,265 (32.7%)	
Acute	4,925 (66.4%)	266 (54.5%)	4,659 (67.3%)	0.222
STE-ACS	2,551 (51.8%)	125 (47.0%)	2,426 (52.1%)	
NSTE-ACS	1889 (38.4%)	117 (44.0%)	1772 (38.0%)	
Other	485 (9.8%)	24 (9.0%)	461 (9.9%)	
Coronary dominance				0.582
Right dominance	5,977 (80.6%)	393 (80.5%)	5,584 (80.6%)	
Left dominance	708 (9.6%)	37 (7.6%)	671 (9.7%)	
Codominance	727 (9.8%)	58 (11.9%)	669 (9.7%)	
SYNTAX score	8 (5-16)	11 (7-19)	8 (5-15)	**<0.001**
Location of treatment				
RCA	2,876 (38.8%)	219 (44.9%)	2,657 (38.4%)	**0.002**
LM	303 (4.1%)	36 (7.4%)	267 (3.9%)	**<0.001**
LAD	3,743 (50.5%)	268 (54.9%)	3,475 (50.2%)	0.023
CX	1863 (25.1%)	127 (26.0%)	1736 (25.1%)	0.583
IM	143 (1.9%)	9 (1.8%)	134 (1.9%)	0.873
Graft	129 (1.7%)	0	129 (1.9%)	
Type of intervention				**<0.001**
Stent	6,936 (93.6%)	417 (85.5%)	6,519 (94.2%)	
DCB	162 (2.2%)	16 (3.3%)	146 (2.1%)	
POBA	314 (4.2%)	55 (11.3%)	259 (3.7%)	
Number of coronary vessels treated				**<0.001**
1 vessel treated	6,136 (82.8%)	361 (74.0%)	5,775 (83.4%)	
2 vessels treated	1,170 (15.8%)	113 (23.2%)	1,057 (15.3%)	
3 vessels treated	106 (1.4%)	14 (2.9%)	92 (1.3%)	
Number of coronary vessels diseased				**<0.001**
1-vessel disease	3,350 (45.2%)	145 (29.7%)	3,205 (46.3%)	
2-vessel disease	2,525 (34.1%)	187 (38.3%)	2,338 (33.8%)	
3-vessel disease	1,537 (20.7%)	156 (32.0%)	1,381 (19.9%)	
Duration of intervention, min	70 (52-96)	89 (63-121)	70 (52-94)	**<0.001**
Contrast volume, mL	210 (152-281)	235 (170-332)	210 (151-280)	**<0.001**

	Total (N = 6,936)	TLR (n = 417, 6.0%)	No TLR (n = 6,519, 94.0%)	
Type of intervention = stent				
Number of stents	1 (1-2)	2 (1-3)	1 (1-2)	**<0.001**
Type of stents				0.267
BMS	61 (0.9%)	5 (1.2%)	56 (0.9%)	
DES 2 (durable)	4,501 (64.9%)	292 (70.0%)	4,209 (64.6%)	
DES 2 (biodegradable)	1917 (27.6%)	91 (21.8%)	1826 (28.0%)	
DES 2 (polymer-free)	150 (2.2%)	9 (2.2%)	141 (2.2%)	
Other	307 (4.4%)	20 (4.8%)	287 (4.4%)	
Total stent length, mm	23 (15-38)	26 (15-48)	23 (15-38)	**0.002**
Minimal stent diameter, mm	3.00 (2.75-3.50)	3.00 (2.50-3.00)	3.00 (2.75-3.50)	**<0.001**
Stent ratio	7.38 (4.67-13.20)	8.67 (4.76-17.45)	7.33 (4.57-13.09)	**<0.001**

Values are median (IQR) or n (%). Statistical significance of univariable regression analyses is shown in **bold**. A total of 7,412 patients were evaluated in this analysis.

BMI = body mass index; BMS = bare metal stent; CRP = C-reactive protein; CX = circumflex artery; DCB = drug-coated balloon; DES = drug-eluting stent; GFR (CKD-EPI) = glomerular filtration rate (chronic kidney disease-epidemiology collaboration); HbA1c = hemoglobin A1c; IM = intermediate artery; LAD = left anterior descending artery; LDL = low-density lipoprotein; LM = left main; NLR = neutrophil-to-lymphocyte ratio; NSTE-ACS = non-ST-segment elevation acute coronary syndrome; NT-proBNP = N-terminal pro-B-type natriuretic peptide; POBA = plain old balloon angioplasty; RCA = right coronary artery; STE-ACS = ST-segment elevation acute coronary syndrome; TLR = target lesion revascularization.

### Association of inflammation with target lesion revascularization

A total of 488 (6.6%) TLR were detected in the median follow-up time of 3.6 years (IQR: 1.2-7.3 years), with 140 of them occurring within the first 30 days after primary intervention. Patients with elevated preinterventional CRP or NLR did not show increased risk of subsequent TLR (Table 2). Leukocyte counts indicated an inverse association with TLR in the univariable analysis with an HR per 1 SD of 0.80 (90% CI: 0.73-0.88; *P* < 0.001) (Table 2). However, after adjustment for clinical and interventional parameters, all investigated inflammatory biomarkers showed no significant association with TLR, neither for short-term nor for long-term follow-up (Table 2). A subgroup analysis of patients with severely elevated inflammatory biomarkers represented by CRP levels above 5.0 mg/dL (n = 498) also suggests similar risk for the occurrence of TLR (5.4% vs 6.7%) compared to patients with lower CRP levels (adjusted HR: 0.96 [95% CI: 0.63-1.45]; *P* = 0.830). In addition, stratification of the total study population into elective (n = 2,487; 33.6%) and acute (n = 4,925; 66.4%) primary interventions did indicate no correlation of inflammatory activation with TLR and 30-day TLR in both subgroups (Table 2). Event curves for CRP showing comparable distributions of TLR in elective and acute primary interventions are illustrated in the Central Illustration.

**Table 2:** Association of CRP, Leukocytes, and NLR With Target Lesion Revascularization

	Crude HR (95% CI)	*P* Value	Adjusted HR (95% CI)	*P* Value
Total cohort, n = 7,412				
TLR, n = 488 (6.6%)				
CRP	0.93 (0.85-1.02)	0.141	0.93 (0.85-1.03)	0.150
Leukocytes	0.80 (0.73-0.88)	**<0.001**	0.88 (0.80-0.98)	0.022
NLR	0.92 (0.84-1.02)	0.115	0.90 (0.82-1.00)	0.056
30-day TLR, n = 140 (1.9%)				
CRP	0.96 (0.81-1.14)	0.622	0.89 (0.75-1.05)	0.177
Leukocytes	1.07 (0.90-1.26)	0.441	1.00 (0.83-1.19)	0.962
NLR	1.15 (0.96-1.36)	0.119	1.03 (0.85-1.24)	0.772
Elective interventions, n = 2,487				
TLR, n = 222 (8.9%)				
CRP	0.86 (0.74-0.98)	0.028	0.88 (0.76-1.02)	0.084
Leukocytes	0.90 (0.79-1.03)	0.138	0.92 (0.80-1.06)	0.243
NLR	0.94 (0.82-1.09)	0.426	0.92 (0.79-1.07)	0.267
30-day TLR, n = 35 (1.4%)				
CRP	1.06 (0.76-1.47)	0.745	1.07 (0.76-1.50)^a^	0.700^a^
Leukocytes	0.98 (0.70-1.37)	0.904	1.01 (0.72-1.40)^a^	0.960^a^
NLR	1.20 (0.87-1.65)	0.259	1.13 (0.81-1.60)^a^	0.468^a^
Acute interventions, n = 4,925				
TLR, n = 266 (5.4%)				
CRP	1.04 (0.92-1.18)	0.524	0.97 (0.85-1.10)	0.605
Leukocytes	0.86 (0.76-0.98)	0.019	0.88 (0.77-1.00)	0.044
NLR	0.95 (0.83-1.09)	0.469	0.89 (0.78-1.03)	0.119
30-day TLR, n = 105 (2.1%)				
CRP	0.90 (0.74-1.10)	0.312	0.83 (0.69-1.01)	0.067
Leukocytes	0.98 (0.81-1.19)	0.847	0.99 (0.82-1.21)	0.947
NLR	1.09 (0.89-1.34)	0.418	1.00 (0.80-1.25)	0.994

Statistical significance of univariable regression analyses is shown in **bold**. Univariable and multivariable Cox proportional hazard model. HR depicts hazard ratios per 1 SD (95% CI). In the multivariable analysis, all investigated inflammatory biomarkers showed no association with TLR whether in the total patient cohort nor after stratification into elective and acute primary interventions after adjustment for age, sex, BMI, indication for intervention, previous revascularization, previous hypertension, previous hyperlipidemia, previous diabetes mellitus type II, GFR (CKD-EPI), coronary dominance, SYNTAX score, type of intervention, LM intervention, number of coronary vessels treated, number of coronary vessels diseased, duration of intervention, and contrast volume. A total of 7,412 patients were evaluated in this analysis.

Abbreviations as in Table 1.

a Cautious interpretation due to overfitting.

**Central illustration:**
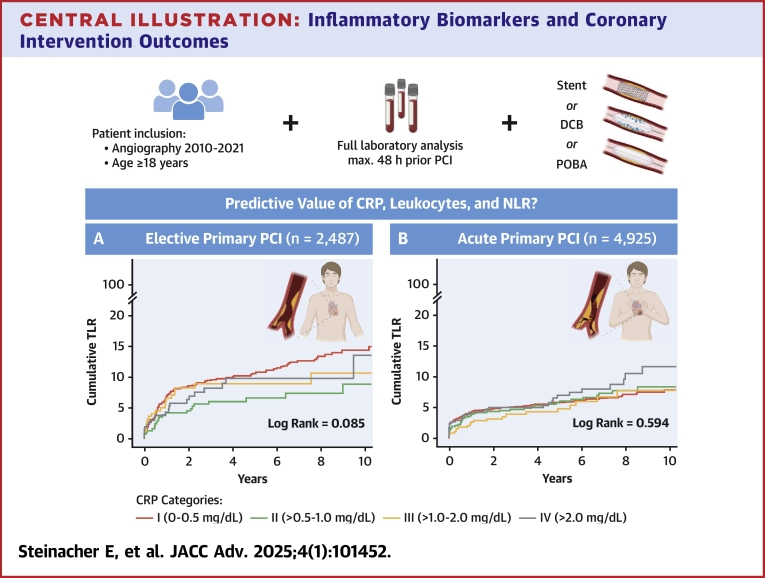
**Inflammatory Biomarkers and Coronary Intervention Outcomes** A total of 7,412 patients undergoing PCI with full standard laboratory analysis within 48 hours prior angiography were screened for adverse events. Time-to-first-event curves for cumulative TLR by categorized CRP show no association with subsequent TLR in patients with elective primary interventions (A) and in patients with acute primary intervention (B). CRP = C-reactive protein; DCB = drug-coated balloons; NLR = neutrophil-to-lymphocyte ratio; PCI = percutaneous coronary intervention; POBA = plain old balloon angioplasty; TLR = target lesion revascularization.

With respect to type of primary intervention, 417 (6.0%) TLR were observed after stenting, 16 (9.9%) after DCB, and 55 (17.5%) after POBA only. Neither CRP nor leukocytes or NLR indicated a risk for TLR in any subgroup. In case of primary intervention with stenting, patients were stratified into PCI with different stent generations. TLR rates were 8.2% (n = 5) for BMS, 6.5% (n = 292) for second-generation durable polymer DES, 4.7% (n = 91) for second-generation biodegradable polymer DES, and 6.0% (n = 9) for second-generation polymer-free DES. Again, no correlation was found for inflammation and TLR when testing separately for different stent types.

### Inflammatory activation in acute stent thrombosis

Patients with 30-day TLR were further screened for revascularization due to acute ST, where 44 (0.6%) patients were detected. Eight (0.3%) TLR occurred after elective primary intervention, and 36 (0.7%) after acute primary intervention. In the total study population, elevated CRP, leukocytes, or NLR during intervention did not indicate an increased risk of subsequent acute ST (Table 3). Similar results were found when stratifying into elective and acute primary interventions and remained negative in multivariable analysis (Table 3).

**Table 3:** Association of CRP, Leukocytes, and NLR With Acute Stent Thrombosis

	Crude HR (95% CI)	*P* Value	Adjusted HR (95% CI)	*P* Value
Total cohort, n = 7,412; acute stent thrombosis, n = 44 (0.6%)				
CRP	1.13 (0.85-1.51)	0.404	1.06 (0.79-1.42)	0.708
Leukocytes	1.24 (0.94-1.65)	0.129	1.05 (0.75-1.45)	0.783
NLR	1.35 (1.00-1.82)	0.049	1.21 (0.88-1.66)	0.243
Elective interventions, n = 2,487; acute stent thrombosis, n = 8 (0.3%)				
CRP	0.97 (0.48-1.95)	0.936	1.14 (0.58-2.24)	0.699
Leukocytes	0.80 (0.40-1.59)	0.515	0.86 (0.42-1.76)	0.684
NLR	1.06 (0.53-2.10)	0.971	1.05 (0.51-2.14)	0.895
Acute interventions, n = 4,925; acute stent thrombosis, n = 36 (0.7%)				
CRP	1.13 (0.82-1.55)	0.466	1.06 (0.76-1.46)	0.743
Leukocytes	1.16 (0.84-1.60)	0.366	1.08 (0.77-1.52)	0.640
NLR	1.35 (0.96-1.90)	0.082	1.27 (0.88-1.83)	0.194

Univariable and multivariable Cox proportional hazard model. HR depicts hazard ratios per 1 SD (95% CI). In the multivariable analysis, all investigated inflammatory biomarkers showed no association with acute stent thrombosis, whether in the total patient cohort nor after stratification into elective and acute primary interventions after adjustment for age, sex, BMI, indication for intervention, previous revascularization, previous hypertension, previous hyperlipidemia, previous diabetes mellitus type II, GFR (CKD-EPI), coronary dominance, SYNTAX Score, type of intervention, LM intervention, number of coronary vessels treated, number of coronary vessels diseased, duration of intervention, and contrast volume. A total of 7,412 patients were evaluated in this analysis.

Abbreviations as in Table 1.

### Mortality and cardiovascular endpoint analysis

**Figure 2:**
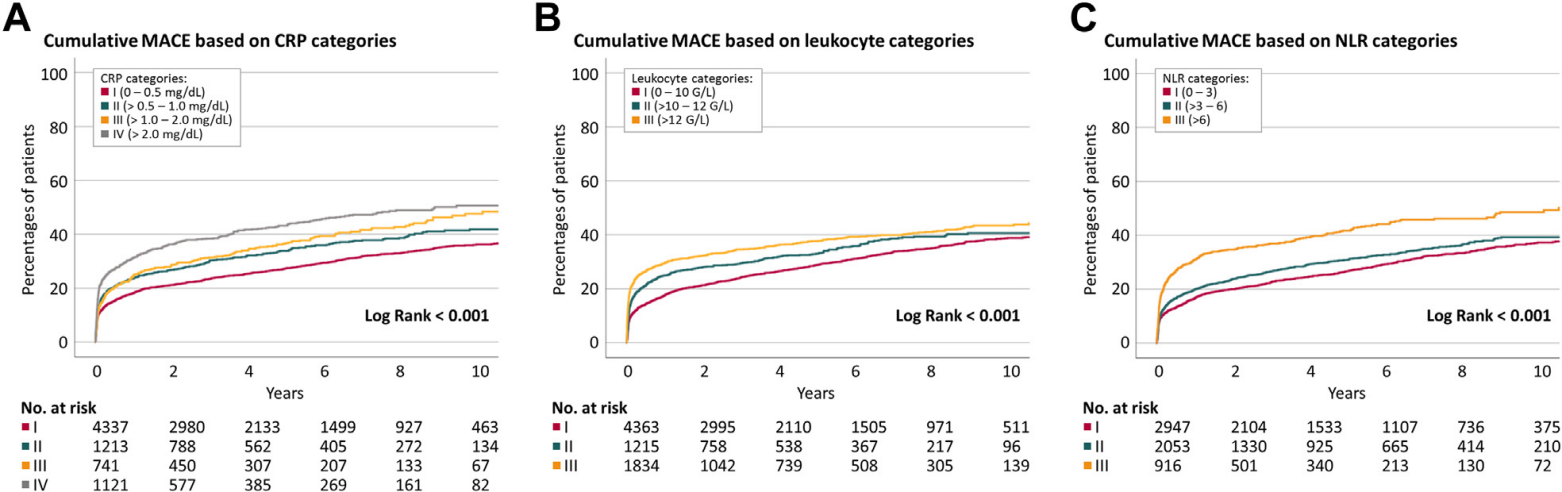
**Time-to-First-Event Curves for Cumulative Major Adverse Cardiovascular Events Through Follow-Up** Results presenting cumulative MACE according to categorized CRP (A), leukocytes (B), and NLR (C) through a median follow-up time of 4.6 years (IQR: 2.1-8.2 years). CRP = C-reactive protein; MACE = major adverse cardiovascular events; NLR = neutrophil-to-lymphocyte ratio.

The overall mortality of the study population was 25.7% (n = 1,906) within a median follow-up time of 4.6 years (IQR: 2.1-8.2 years). A total of 950 (12.8%) patients died of cardiovascular causes and 2,456 (33.1%) patients reached 3-point MACE during follow-up. All investigated inflammatory biomarkers showed strong associations with mortality, cardiovascular mortality, and MACE rates in univariable Cox proportional hazard analysis as presented in Table 4. These associations remained highly significant after comprehensive adjustment for clinical and interventional variables (Table 4). Corresponding time-to-event curves illustrating MACE according to CRP, leukocyte, and NLR categories are shown in Figure 2. Analogous curves for mortality and cardiovascular mortality are illustrated in Supplemental Figure 1.

**Table 4:** Association of CRP, Leukocytes, and NLR With Mortality and 3-Point MACE

	Crude HR (95% CI)	*P* Value	Adjusted HR (95% CI)	*P* Value
Mortality, n = 1,906 (25.7%)				
CRP	1.57 (1.50-1.64)	**<0.001**	1.35 (1.29-1.41)	**<0.001**
Leukocytes	1.15 (1.10-1.21)	**<0.001**	1.28 (1.22-1.34)	**<0.001**
NLR	1.62 (1.54-1.70)	**<0.001**	1.36 (1.29-1.43)	**<0.001**
Cardiovascular mortality, n = 950 (12.8%)				
CRP	1.57 (1.48-1.67)	**<0.001**	1.30 (1.22-1.38)	**<0.001**
Leukocytes	1.37 (1.29-1.46)	**<0.001**	1.44 (1.36-1.53)	**<0.001**
NLR	1.78 (1.66-1.91)	**<0.001**	1.44 (1.34-1.56)	**<0.001**
3-point MACE, n = 2,456 (33.1%)				
CRP	1.26 (1.22-1.31)	**<0.001**	1.14 (1.09-1.19)	**<0.001**
Leukocytes	1.18 (1.14-1.23)	**<0.001**	1.11 (1.07-1.16)	**<0.001**
NLR	1.20 (1.15-1.25)	**<0.001**	1.08 (1.03-1.13)	**<0.001**

Statistical significance of univariable regression analyses is shown in **bold**. Univariable and multivariable Cox proportional hazard model. HR depicts hazard ratios per 1 SD (95% CI). In the multivariable analysis, all investigated inflammatory biomarkers remained significantly associated with mortality, cardiovascular mortality, and 3-point MACE after adjustment for age, sex, BMI, indication for intervention, previous revascularization, previous hypertension, previous hyperlipidemia, previous diabetes mellitus type II, GFR (CKD-EPI), coronary dominance, SYNTAX Score, type of intervention, LM intervention, number of coronary vessels treated, number of coronary vessels diseased, duration of intervention, and contrast volume. A total of 7,412 patients were evaluated in this analysis.

Abbreviations as in Table 1.

Neither all-cause mortality (TLR: 23.8% vs no TLR: 25.9%; *P* = 0.309) nor cardiovascular mortality (TLR: 11.5% vs no TLR: 12.9%; *P* = 0.359) was found to be significantly different between patients with subsequent TLR and patients with favorable interventional results. However, patients with TLR showed significantly higher MACE rates (TLR: 53.9% vs no TLR: 31.7%; *P* < 0.001).

## Discussion

In this unselected study cohort comprising 7,412 patients undergoing PCI, we were able to report 3 main findings. First, systemic inflammation reflected by elevated CRP, leukocytes, or NLR is not associated with the incidence of TLR after either acute or elective PCI. Second, inflammatory activation is not related to 30-day ST. Third, elevated inflammatory parameters are, however, strongly and robustly associated with all-cause mortality, cardiovascular mortality, or MACE rates after PCI.

### Inflammation and target lesion revascularization

Inflammation is the major pathophysiological pathway in all stages of atherosclerosis. Mediators of inflammation regulate atheroma initiation, plaque destruction, and healing processes. Additionally, by inducing procoagulant factors and altering the vessel wall, inflammatory activation substantially promotes platelet aggregation.^3^

The large, unselected PCI registry examined in this study, comprising a total of 7,412 patients, proved to be representative of a general PCI cohort in its baseline characteristics with a median patient age of 64 years, moderately overweight subjects with a median BMI of 27.1 kg/m^2^, and approximately 75% of all patients being male. In our study cohort, we identified a total of 488 patients with TLR after a median follow-up of 3.6 years. Based on the assumption that inflammation might contribute to worse interventional outcomes, patients with elevated baseline inflammatory parameters are often postponed from PCIs. Although the influence of inflammation on PCI outcomes is suspected in clinical practice, our analysis shows no significant association between elevated baseline inflammatory parameters and TLR, neither at long-term nor at 30-day follow-up. Even after stratification into elective and acute interventions, no correlation between systemic inflammation and adverse angiographic outcomes could be observed.

Previous research in this area has been underpowered and limited to BMS and early-generation DES. In 2009, a prospective analysis including a total of 2,691 patients demonstrated an association of CRP with death, myocardial infarction, and ST after early-generation DES implantation and a median follow-up time of 3.9 years.^14^ Smaller studies followed, suggesting a clinical and angiographic predictive value of increased baseline CRP, particularly in relation to thrombotic events.^13^^,^^15^ One analysis showed preferential results for DES implantation compared with BMS in ST-segment elevation acute coronary syndrome patients in relation to ST in the presence of elevated high-sensitive CRP.^17^ In 2015, an all-comer PCI registry with the longest follow-up time of 10 years found a significant association of CRP with death and myocardial infarction in patients after first-generation DES implantation.^16^ However, in the context of advancing interventional cardiology and improving stent technology, significant progress has been made.

DES have almost completely replaced BMS in clinical practice. DES release drugs that inhibit excessive tissue growth due to the locally induced inflammatory response in the vessel wall upon intervention.^8^^,^^9^ Currently used second-generation DES with improved design show decreased risk for ST compared with first-generation DES.^10^ New-generation DES differ particularly in their polymer coating. While a durable polymer coating was originally used, the recent trend is toward biodegradable polymer coating to polymer-free DES.^18^ New generations of DES show lower rates of myocardial infarction and ST.^11^

In our study population, subgroup analyses for the use of stents, POBA, or DCB showed no significant association between systemic inflammation and TLR. When testing separately for different stent types, we again found no correlation between inflammation and adverse angiographic events. Notably, primarily second-generation DES were analyzed in this study, and only approximately 5% of other stents including BMS and first-generation DES. We interpret that these findings derive from the inability to correlate the localized stimulus of the intervention with systemic markers. Our baseline characteristics indicate that procedural factors, especially factors of complexity such as SYNTAX score, location of treatment, number of diseased vessels, and stent ratio, as well as the extent of preexisting atherosclerosis, are more significantly associated with the risk of TLR.

### Inflammation and acute stent thrombosis

Acute ST is a major cause of coronary stent failure and a potentially fatal complication after PCI. Although they have become rare due to modern interventional technologies, they remain a feared complication for interventionalists.^19^ In our patient population, a total of 44 (0.6%) patients were revascularized due to acute ST within the first 30 days after PCI. According to our analyses, the occurrence of 30-day ST does not seem to be associated with increased baseline inflammatory parameters, neither in acute nor in elective interventions.

Data on the interaction between systemic inflammation and acute ST are limited, as discussed in the above paragraph. Inflammation due to infections, autoimmune diseases, and certain blood disorders increases the risk of thrombi, suggesting a nexus between inflammation and thrombosis.^20^ An optical coherence tomography imaging study has suggested that local signs of inflammation in the coronary artery correlate with increased incidence of ST.^21^ In our study, data suggest that systemic inflammation has no association with acute ST leading to the assumption that local vascular inflammation has a much more significant position. Other factors including interruption of dual antiplatelet therapy or the use of undersized stents, appear to play a more significant role in provoking acute ST.^22^ Elevated baseline level of inflammation, in contrast, does not seem to pose a threat to intervention and accordingly should not delay PCI.

### Inflammation and cardiovascular events

Previous studies demonstrated the predictive value of inflammatory activation for cardiovascular events in primary and secondary prevention.^23^^,^^24^ Our data are completely in line with these findings. In our cohort, all investigated inflammatory parameters including CRP, leukocytes, and NLR are strongly and robustly associated with all-cause mortality, cardiovascular mortality, and MACE rates after PCI.

Currently, there is limited understanding of the role of inflammation in CAD patients, but therapeutic inhibition of inflammation to reduce the risk of cardiovascular events is increasingly coming into focus.^25^ Ridker et al recently showed that despite established statin therapy, residual risk for inflammation appears to be more strongly associated with cardiovascular events than residual risk for cholesterol.^26^ Three randomized trials on the use of colchicine and canakinumab as add-on therapy in CAD have already shown promising results.^27^, ^28^, ^29^ The combination of lipid-lowering and anti-inflammatory therapy may represent the future standard therapy for CAD and is being evaluated in ongoing clinical trials.^30^ Our data emphasize this approach as well, especially in secondary prevention after coronary intervention has already been performed.

### Study limitations

There are several limitations to the present study that should be considered when interpreting the findings.

This study is a single-center study with its inherent limitations that included only patients from the local high-frequency cardiac catheterization laboratory of the Medical University of Vienna. In addition, the real-world nature of the study cohort implies that only few patients with acute infections and fever were included, as they are less frequently assigned to elective PCI in clinical practice as described above. This selection bias should be considered for generalizability, and further studies with specific evaluation of safety of PCI in these situations are necessary.

There is a residual possibility that endpoints were not recorded if patients presented at hospitals outside Vienna or private clinics. However, as endpoints were collected from all hospitals of the Viennese Hospital Association providing the majority of local patient care, it is assumed that these are only a minority of individual cases, which are negligible in our large sample with adequate power.

Furthermore, the heterogeneity of the study's all-comer design may pose a challenge in identifying specific associations, although this is precisely what reflects the reality of patients with PCI. As this is an observational study, it is difficult to establish causal conclusions. Further randomized controlled trials are needed to derive evidence-based recommendations for optimal patient care in clinical practice. Despite these limitations, we aim to highlight the large study cohort, long follow-up period, and unselected patient sample, which contribute to the robustness of the present data and the minimization of bias.

## Conclusions

Our study shows no significant association between systemic inflammation and adverse interventional outcomes, although an impact of inflammation on coronary outcomes is suspected. CRP, leukocytes, and NLR do not appear to have a significant impact on TLR or acute ST in patients undergoing PCI. Instead, procedural factors, complexity of the intervention, and the extent of preexisting atherosclerosis appear to be more important in their association with TLR.

In contrast, inflammatory activation is strongly associated with mortality and the occurrence of adverse cardiovascular events after PCI. Present findings highlight the increasing importance of investigating anti-inflammatory therapeutic options in addition to traditional standard lipid-lowering therapy in the treatment of CAD, particularly in secondary prevention after PCI. According to our analysis, elevated baseline inflammatory parameters should, however, not delay PCI in the era of second-generation DES.

Perspectives **COMPETENCY IN MEDICAL KNOWLEDGE 1:** Inflammatory biomarkers are reliable predictors of adverse cardiovascular events in patients suffering from coronary artery disease.

**COMPETENCY IN MEDICAL KNOWLEDGE 2:** In this analysis of 7,412 patients undergoing PCI, systemic inflammation was strongly associated with mortality and 3-point MACE. However, it did not appear to affect interventional outcomes including TLR and acute ST.

**TRANSLATIONAL OUTLOOK:** These results indicate that elevated baseline inflammatory biomarkers should not delay PCI in the era of second-generation DES.

## Funding support and author disclosures

The authors have reported that they have no relationships relevant to the contents of this paper to disclose.
