# Supplementary material for: Routinely Available Inflammatory Biomarkers Are Not Associated With Target Lesion Revascularization After Coronary Intervention
Source: JACC Adv. 2025 Jan 22;4(1):101452. doi: 10.1016/j.jacadv.2024.101452 (PMC11822304; doi:10.1016/j.jacadv.2024.101452)

**Supplemental Appendix**

**Suppl. Figure 1.** Time-to-first-event curves for cumulative all-cause and cardiovascular mortality through follow up……………………………………………………………………2

**Suppl. Figure 1. Time-to-first-event curves for cumulative all-cause and cardiovascular mortality through follow up.** Results presenting cumulative all-cause mortality (A-C) and cardiovascular mortality (D-F) according to categorized CRP (A, D), leukocytes (B, E) and NLR (C, F) through a median follow up time of 4.6 years (IQR 2.1 – 8.2). CRP = C-reactive protein, NLR = neutrophil-to-lymphocyte ratio, No. = number.


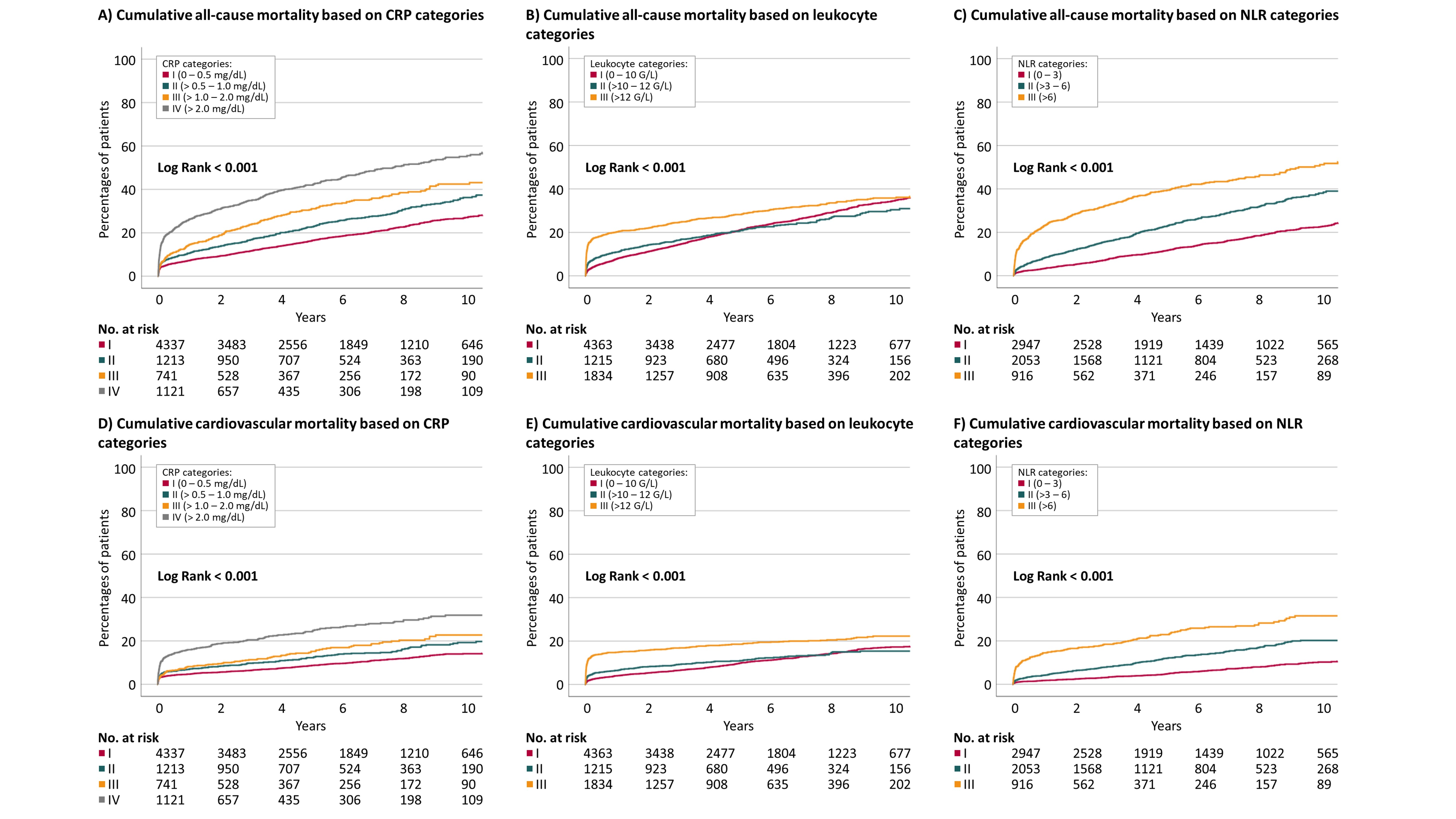

Supplement: Supplemental Figure 1 [file mmc1.docx]
